# Transformation of a prostatic adenocarcinoma into squamous cell carcinoma after luteinizing hormone-releasing hormone (LHRH) agonist and radiotherapy treatment

**DOI:** 10.11604/pamj.2019.34.125.19421

**Published:** 2019-11-01

**Authors:** Hamza Ichaoui, Sonia Ben Nasr, Faten Gargouri, Aref Zribi, Amine Hermi, Sana Fendri, Mehdi Balti, Jihen Ayari, Ramzi Khiari, Issam Msakni, Nada Mansouri, Samir Ghozzi, Abderrazek Haddaoui

**Affiliations:** 1 Université de Tunis El Manar, Faculté de Médecine de Tunis, 1007, Tunis, Tunisie; 2 The Military Hospital of Tunis, Department of Urology, Montfleury 1008, Tunis, Tunisia; 3 Université de Tunis El Manar, Faculté de Médecine de Tunis, 1007, Tunis, Tunisie; 4 The Military Hospital of Tunis, Department of Medical Oncology, Montfleury 1008, Tunis, Tunisia; 5 Université de Tunis El Manar, Faculté de Médecine de Tunis, 1007, Tunis, Tunisie; 6 The Military Hospital of Tunis, Department of Pathology, Montfleury 1008, Tunis, Tunisia

**Keywords:** Prostate, squamous cell carcinoma, hormonotherapy

## Abstract

Squamous cell carcinoma of the prostate is rare and represents 0.5% to 1% of prostatic carcinomas. Transformation of prostatic adenocarcinoma into squamous cell carcinoma after LH-RH agonist intake has been reported in only 8 cases in the literature. To our knowledge, our case is the second pure squamous cell carcinoma observed after hormonotherapy and radiotherapy. We reported a case of a patient with prostatic adenocarcinoma treated by radical prostatectomy followed by radiotherapy. Eleven years later, he had a vesical recurrence of prostatic adenocarcinoma. Our patient had an endoscopic resection followed by injections of Triptorelin. Six months later, he developed a local recurrence of a squamous cell carcinoma.

## Introduction

Prostatic Squamous Cell Carcinoma (SCC) is a rare tumor with aggressive nature and represents 0.5% to 1% of prostatic carcinomas [1,2]. Therefore, clinical presentation, symptoms, treatment and prognosis were reported only in case reports. SCC has a poor response to conventional treatment and results in poor prognosis because of early metastasis to the bone, liver and lungs with an average survival of 14 months [1,2]. Transformation of prostatic adenocarcinoma to SCC after injection of LH-RH agonist or radiotherapy (RT) have been reported in the literature in few cases. We reported a case of prostatic SCC developed in a patient with prostatic ADK following hormonotherapy (HT).

## Patient and observation

**Figure 1:**
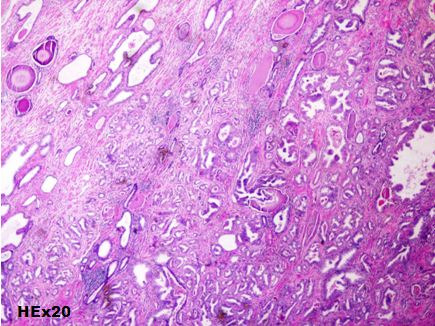
Gleason 6 (3+3) prostatic ADK: individual well-formed glands

**Figure 2:**
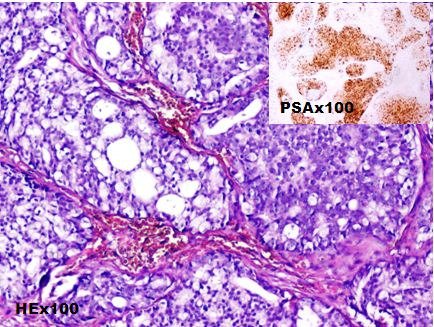
Vesical localization of a prostatic ADK (PSA +): large irregular cribriform massives

**Figure 3:**
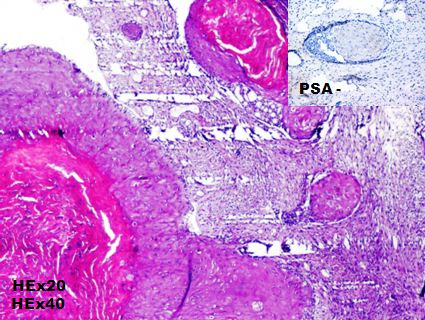
Bladder squamous cell carcinoma: massive of atypical squamous cells (PSA-) centered by keratin pearls

A 71-year-old man consulted for a clinically suspected prostate hypertrophy with a serum Prostate-specific antigen (PSA) level within the reference range of 2.7ng/ml (normal: 0-4 ng/mL). Transrectal ultrasound guided needle prostatic biopsy concluded to Gleason 6 (3+3) prostatic adenocarcinoma. Prostatic MRI showed a low signal intensity mass in peripheral zone. Diffusion weighted image showed a high signal intensity mass in peripheral zone without extracapsular extension. Bone scan and Chest, Abdomen and pelvis CT-scan didn't show metastasis. A radical prostatectomy was performed and pathological examination concluded to a Gleason 6 ADK (3 + 3) classified pT2aN0M0 (Figure 1). HT using LHRH analogue (Triptorelin) and antiandrogen agent (bicalutamide) were started, and serum PSA level gradually decreased to a nadir of 0.04ng/ml. The patient had a biological recurrence (PSA at 0.52) after 2 years with a doubling time greater than 12 months. He had a salvage RT (66Gy) and the PSA level didn't exceed 0.08ng/ml over a period of 4 years. Nine years after treatment, patient complained of hematuria with a rise in PSA level between 0.33 and 0.59ng/ml over a period of three years. A cystoscopy objectified a peri-cervical mass of 2 cm whose resection concluded to a vesical localization of a prostatic ADK (Figure 2). HT (Triptorelin and Cyproterone acetate) was done. Six months later, hematuria recurred and cystoscopy objectified a bladder recurrence. Histopathological examination concluded to a bladder squamous cell carcinoma (Figure 3). The patient refused total cystectomy. He had Gemcitabin and Cisplatinum chemotherapy (CT) combined with Triptorelin with partial response after three cycles of CT and good tolerance. The patient is still under treatment.

## Discussion

Squamous cell carcinoma of the prostate is a rare tumor (0.5 to 1% of all prostate cancers) with aggressive nature and more frequent extension beyond the prostate gland [2,3]. The average age of onset is 68 years with extremes ranging from 42 to 85 years [2,4]. It differs from common ADK in its therapeutic response and prognosis. This type of tumor has a poor response to conventional treatment resulting in poor prognosis. Due to high degree of malignancy, SCC of the prostate generally metastasizes early to the bone, liver, lungs, and lymph nodes. The bone metastases are usually osteolytic rather than osteoblastic [1,5]. The prostate-specific antigen (PSA) test and Gleason score are of limited value in epidermoid carcinoma diagnosis, and the median survival rate after diagnosis is estimated at 14 months [5-8]. About half of cases occur after the management of prostatic ADK with HT and/or RT. However, they are often associated with an ADK component. Sometimes, patients have no history of prostatic disease, or other locations of SCC, it is then a primitive form of prostate SCC [2,9]. The histological features of prostatic SCC include the presence of cellular metaplasia and local invasion with signs of squamous differentiation: keratinization, the presence of squamous beads and/or many distinct intercellular bridges [10]. It may be challenging to distinguish between SCC and non-neoplastic squamous metaplasia, which may be secondary to infarct, acute or chronic prostatitis, granulomatous prostatitis due to BCG therapy, estrogen therapy or pelvic irradiation [11]. Epidermoid differentiation in prostate cancer can be found either in pure form, or associated with ADK, urothelial carcinoma or sarcoma contingent. The absence of ADK component distinguishes pure SCC from adenosquamous carcinoma [9]. The histogenesis of SCC has always been controversial. The origin may be prostatic or bladder urethral squamous cell, prostatic acini metaplasia, or squamous metaplasia of a prostatic tumor [1,3].Transformation of ADK to SCC occurred secondary to RT or HT. Most case reports of squamous transformation occurring after RT or HT tended to be associated with high grade ADK. Extensive squamous metaplasia, pluripotent stem cells capable of multidirectional differentiation or metaplastic transformation of adenocarcinoma were reported to be possible origins [1]. Lager *et al* [12] reported SCC developed due to adverse stimuli affecting columnar cells causing them to express normal prostatic antigen such as PSA and prostatic acid phosphatase, although retaining the ability to produce keratin resulting in no elevated serum PSA even in a metastatic disease context.

Given its possible multiple origins, it may be challenging to decide whether the epidermoid component developed from a divergent differentiation of ADK after RT or HT, or from a squamous differentiation of carcinoma to Transitional cells, or it is just a primitive epidermoid carcinoma, called “De Novo” [1]. Imaging diagnosis of SCC of the prostate is challenging because of its rarity and lack of well-established imaging characteristics. Differential diagnosis of a rapidly growing prostate mass with aggressive nature includes recurred adenocarcinoma and small cell differentiation. If a rapid growth of a prostate mass with a history of RT or HT was noted, squamous transformation of prostate cancer is possible [1]. The treatment is controversial. Various approaches including surgical intervention, CT and RT have been implemented without durable response [1]. In our study, we reported a case of pure SCC that appeared six months after injection of an LH-RH agonist (Triptorelin) and eight years after external beam RT. With did a review of the literature focusing on epidermoid carcinoma that occurred after a radio or hormonotherapy (Table 1). Over seventy cases of prostatic cancer with epidermoid differentiation were reported in English literature [2]. Forty of these cases were caused by the transformation of prostatic ADK into SCC after RT and/or HT. Carcinoma was purely epidermoid in 8 cases and adenosquamous in 32 cases [1,4-6, 9,13-20]. Only seven cases were reported after injection of an LH-RH agonist, among which, only one case was a pure epidermoid carcinoma [5]. To the best of our knowledge, our patient is the second case presenting a pure epidermoid carcinoma resulting from the transformation of a prostatic ADK after injection of a LH-RH agonist. Brazilis *et al* [6] reported the first case of prostatic ADK transformation into epidermoid carcinoma after injection of an LH-RH agonist. They recommended the report of any other cases to prove the incrimination of LH-RH agonists into the histogenesis of squamous cell carcinomas. Our case should contribute in helping pathologists, oncologists, radiologists and urologists to better understand the nature and pathogenesis of this aggressive tumor to improve its management.

**Table 1:** Published cases about transformation of prostatic adenocarcinoma into SCC after HT and RT

	Pure epidermoid carcinoma	Adenosquamous carcinoma	Prior treatment
Williams MJ *et al.* (1956)	--	2 cases	Orchidectomy + Stilbestrol
Wernert *et al.* (1990)	--	2 cases	estrogenotherapy
Devaney *et al.* (1991)	--	1 case	estrogenotherapy (Stilbestrol)
Brazilis *et al.* (1995)	--	1 case	-LHRH analogue (Leuprorelin) + Flutamide
Miller VA *et al.* (1995)	1 case	--	brachytherapy
Bassler Tj *et al*. (1999)	--	5 cases	HT and/or RT
Helal M *et al.* (2000)		2 cases	Radiotherapy
Mohan *et al*. (2003)	1 case	--	Bilateral orchidectomy + RT
Parwani et al. (2004)	3 cases. Note: Authors didn’t specify the treatment received for these three patients.	18 cases	-LHRH analogue (Leuprorelin) : 4 cases - Estrogenes: 2 cases -4 cases occurred after RT -1 case occurred after RT+HT the remaining cases were not specified
John TT *et al.* (2005)	1 case	--	Bilateral Orchidectomy
Arva *et al.* (2011)	1 case	--	brachytherapy
AL Quasim *et al.* (2014)	1 case	--	-LHRH analogue (Leuprorelin)
Jihyun Lee (2019)	--	1 case	LHRH analogue (goserelin) and antiandrogen agent (bicalutamide)
Total	8 cases	32 cases	

## Conclusion

Prostatic SCC is rare. Transformation of a prostatic ADK into SCC after HT with a LH-RH agonist or RT is rarer. As the number of patients treated with LH-RH agonists is growing, it will be interesting to understand the histogenesis of this tumor to improve therapy and prognosis.

## Competing interests

The authors declare no competing interests.
